# Variation of sensitivity of *Trypanosoma evansi* isolates from Isiolo and Marsabit counties of Kenya to locally available trypanocidal drugs

**DOI:** 10.1371/journal.pone.0281180

**Published:** 2023-02-02

**Authors:** Raymond E. Mdachi, Kennedy O. Ogolla, Joanna E. Auma, Florence N. Wamwiri, Richard K. Kurgat, Kennedy B. Wanjala, Lawrence G. Mugunieri, Phylis M. Alusi, Judith K. Chemuliti, Phoebe W. Mukiria, Sylvance O. Okoth

**Affiliations:** 1 Biotechnology Research Institute, KALRO, Kikuyu, Kenya; 2 East African Science and Technology Commission (EASTECO)\East African Community, Kigali, Rwanda; Beni Suef University Faculty of Veterinary Medicine, EGYPT

## Abstract

Trypanocidal resistance is a major cause of treatment failure. This study evaluated the sensitivity of *Trypanosoma evansi* field isolates collected from Marsabit and Isiolo counties, Kenya. A total of 2,750 camels were screened using parasitological tests for trypanosomes. Of the screened camels, 113 tested positive from which 40 *T*. *evansi* isolates were tested using the single dose mice sensitivity test. Five treatment groups each comprising of 6 mice were inoculated intraperitoneally with 1x10^5^ trypanosomes of each isolate and treated 24 hours later with isometamidium chloride at 1 mg/kg, homidium chloride at 1mg/kg, diminazene aceturate at 20 mg/kg and quinapyramine sulphate & chloride at 1 mg/kg. The fifth group was left untreated (positive control). The mice were monitored daily for 60 days. A survey on camel owners’ practices that influence development of resistance to trypanocidal drugs was then conducted. Results indicated presence of drug resistance in all the 7 study sites that had infected camels. Seven of the isolates tested were resistant to diminazene aceturate whereas, 28, 33 and 34 were resistant to isometamidium chloride, quinapyramine sulphate & chloride and homidium chloride, respectively. Seven (17.5%) isolates of the 40 tested were sensitive to all 4 drugs, whereas, 7.5%, 10%,55% and 10% were resistant to 1,2,3 and 4 drugs, respectively. The prevalence of multiple drug resistance was 75%. Survey data indicated that camel management practices influenced the prevalence and degree of drug resistance. In conclusion, the multiple drug resistance observed in the two counties may not be an indication of total trypanocidal drug failure. Judicious treatment of confirmed trypanosomiasis cases with correct dosage would still be effective in controlling the disease since the observed resistance was at the population and not clonal level. However, integrated control of the disease and the vectors using available alternative methods is recommended to reduce drug use.

## Introduction

Trypanosomiasis (*Surra*) caused by *T*. *evansi* is one of the most important diseases of camels in the camel keeping belt in the horn of Africa. *Surra* is endemic in areas where camels are kept and occurs with varying degrees of prevalence causing significant economic losses with far reaching implications on food and nutrition security of pastoral communities [1]. *T*. *evansi* is transmitted mechanically by biting flies of the genera *Tabanus*, *Lyperosia*, *Stomoxys*, *Atylotus* [2] and *Hipobosca* [3–6]. *Surra* presents with clinical signs such as fluctuating fever, anemia, weight loss, oedema of ventral regions, lymphadenopathy and sudden death [2,7,8]. It is the most widely distributed of the pathogenic animal trypanosomiasis [9,10] and is the most important single cause of economic losses in camels causing morbidity of up to 30% and mortality of around 3% [11,12]. In Kenya, trypanosomiasis has been identified as the most important disease of camels [13]. The disease is endemic in camel-keeping areas where it occurs with varying degrees of prevalence [14,15]. Camels are susceptible to the different types of trypanosome species; *T*. *evansi*, *T*. *brucei* and *T*. *congolense*. While *T*. *evansi* infections, transmitted by non-tsetse biting flies occur beyond the limits of tsetse fly distribution, the latter two trypanosome species are found mainly in tsetse infested areas [10]. Existence of different levels of virulence in *T*. *evansi* isolates from Kenya has been observed [16].

Management of *T*. *evansi* largely depend on treatment with different trypanocidal drugs; polysulfonated naphthylamine, quinapyramine sulphate & chloride, melarsomine, homidium salts and diminazene aceturate [1]. In areas where *T*. *congolense* infections occur, isometamidium chloride is the preferred drug of choice. The therapeutic efficacy of polysulfonated naphthylamine, quinapyramine, diminazene and isometamedium has been evaluated in naturally *T*. *evansi*-infected buffaloes by Singh and Joshi [17]. Comparative evaluation of all the four drugs indicated that quinapyramine and isometamedium were good curative agents but prophylactically quinapyramine proved better than isometamidium [17]. However, due to high cost, inaccessibility and unavailability of these trypanocidals in remote areas where camels are kept, the majority of the pastoralists resort to use of an assortment of trypanocidals, antibiotics and ethno-veterinary (herbal) products in the treatment of *Surra*. Trypanocidals have been in use for more than half a century and there have been increased reports of development of drug resistance (see Kasozi *et al*. [18]). Geerts & Holmes [19] estimated that 35 million doses of trypanocides are administered every year in sub-Saharan Africa, with isometamidium, ethidium bromide and diminazene aceturate representing 40%, 26% and 33% respectively.

Melarsomine has been found to mitigate the problem of drug resistance to polysulfonated naphthylamine and quinapyramine [20] but costs more than these traditional drugs [21] which has limited its adoption. A recent survey conducted among some camel keepers in the Laisamis and Loglogo areas in Marsabit County in Kenya showed that 71.4% of camel owners used conventional antibiotics, while 37.1% used herbal concoctions as alternative drugs and remedy for the treatment of *Surra* owing to the high cost of melarsomine [15]. The second phase of the survey undertaken in the neighboring camel keeping areas in Isiolo showed that isometamidium chloride and homidium chloride were also used to treat the disease, while 61.4% and 68.2% of the pastoralists from Kulamawe (Isiolo) used quinapyramine and homidium chloride, respectively, to treat camel trypanosomiasis [15]. Consistent findings of high trypanocidal drug use were also reported in a similar survey conducted in Isiolo and Marsabit counties [22]. The high trypanocidal drug use is taking place against a background of privatized veterinary services that resulted in inadequate professional veterinary service providers and increase of unskilled community animal health service providers in the remote areas [23–25]. Therefore, the wide use of trypanocidal drugs by unskilled persons as well as treatment undertaken by camel keepers [26] could potentially compromise the efficacy of treatments and contribute to the development of drug resistance.

It is important to understand that drug resistance does not imply an end to chemotherapy. Studies have shown that resistant trypanosome populations vary greatly in the sensitivity of individual trypanosomes in that population [27–29]. In addition, Ndung’u et al. [30] demonstrated the existence of variations in virulence and sensitivity of *T*. *b*. *rhodesiense* cloned stabilates from one isolate. This suggests that drug resistant populations can be effectively treated by increasing the dosage or through multiple treatment [17,31], or through combination of different synergistic trypanocides [32]. Several factors have been considered as contributing to development of drug resistance such as prolonged exposure of trypanosomes to sub-therapeutic drug levels, thereby favoring the selection of drug-resistant populations [33,34], immunological status of the host [35], high incidence of trypanosomiasis [36] and erratic treatments with prophylactics among others [37–39]. Exposure of trypanosomes to sub therapeutic drug levels has been attributed to incorrect body weight estimation, failure to calculate correct dosage, deliberate under dosing, poor drug preparation and administration [26]. Inappropriate use of trypanocidals by farmers such as self-treatment of sick camels by farmers, wrong route of administration, wrong reconstitution and dosages have been associated with development of drug resistance which has been reported for diminazene aceturate, Isometadidium chloride and other trypanocides in East Africa (See Kasozi et al. [40] for detailed review of farmers’ practices contributing to trypanocidal resistance).

Instability of resistance to trypanocidal compounds in trypanosome populations has been reported [34,38,41]. However, Mulugeta *et al*. [42] showed that the phenotype of multiple drug-resistant *T*. *congolense* remained stable over a period of four years. In *T*. *evansi*, drug resistance has been reported in several type A strains originating from Africa, Asia and Latin America [43,44]. Some Chinese strains appear to be innately resistant to the phenanthridine class of drugs [45,46]. Decrease in virulence and loss of fitness in drug resistant *T*. *evansi* has been reported [47,48]. This loss of fitness has been suggested to contribute immensely in the epidemiology of drug resistance [36,49].

Earlier studies in Marsabit and Isiolo counties of Kenya revealed that 73.2% of the isolates collected from camels showed resistance to isometamidium chloride, 31.6% of the isolates showed resistance to diminazene aceturate while 89.5% of the isolates exhibited resistance to quinapyramine sulphate & chloride at the respective doses tested [15]. In this same study the prevalence of multiple drug resistance (89.0%) was more than two fold the prevalence of resistance (31.5%) observed in *T*. *evansi* isolates collected 15 years earlier [50] in the same region. This indicated that the prevalence of drug resistance is not static and changes with time and in space, perhaps due to a number of socioeconomic factors associated with drug use by different communities. Therefore, the present study aimed to determine variation of sensitivity of *T*. *evansi* population in selected sites in Marsabit and Isiolo counties and effect of various management practices and disease prevalence on this variation.

## Materials and methods

### Ethical approval and informed consent

The study was approved by the Kenya Agricultural and Livestock Research Organization (KALRO)-Institutional Animal Care and Use Committee (KALRO-IACUC) while the survey of camel owners was approved by Kenya Agricultural and Livestock Research Organization Scientific Review Committee. The camel keepers who participated in this study signed a written informed consent form before camel sampling and questionnaire administration. The process entailed providing the camel keepers with adequate information about the study, outlining the possible benefits and consequences of participating in the study, responding to the farmers’ questions, making it clear that they were free to discontinue the interview at any point, providing adequate time for them to make informed decision and then getting their permission before proceeding with the study.

### Sample collection sites

**Fig 1:**
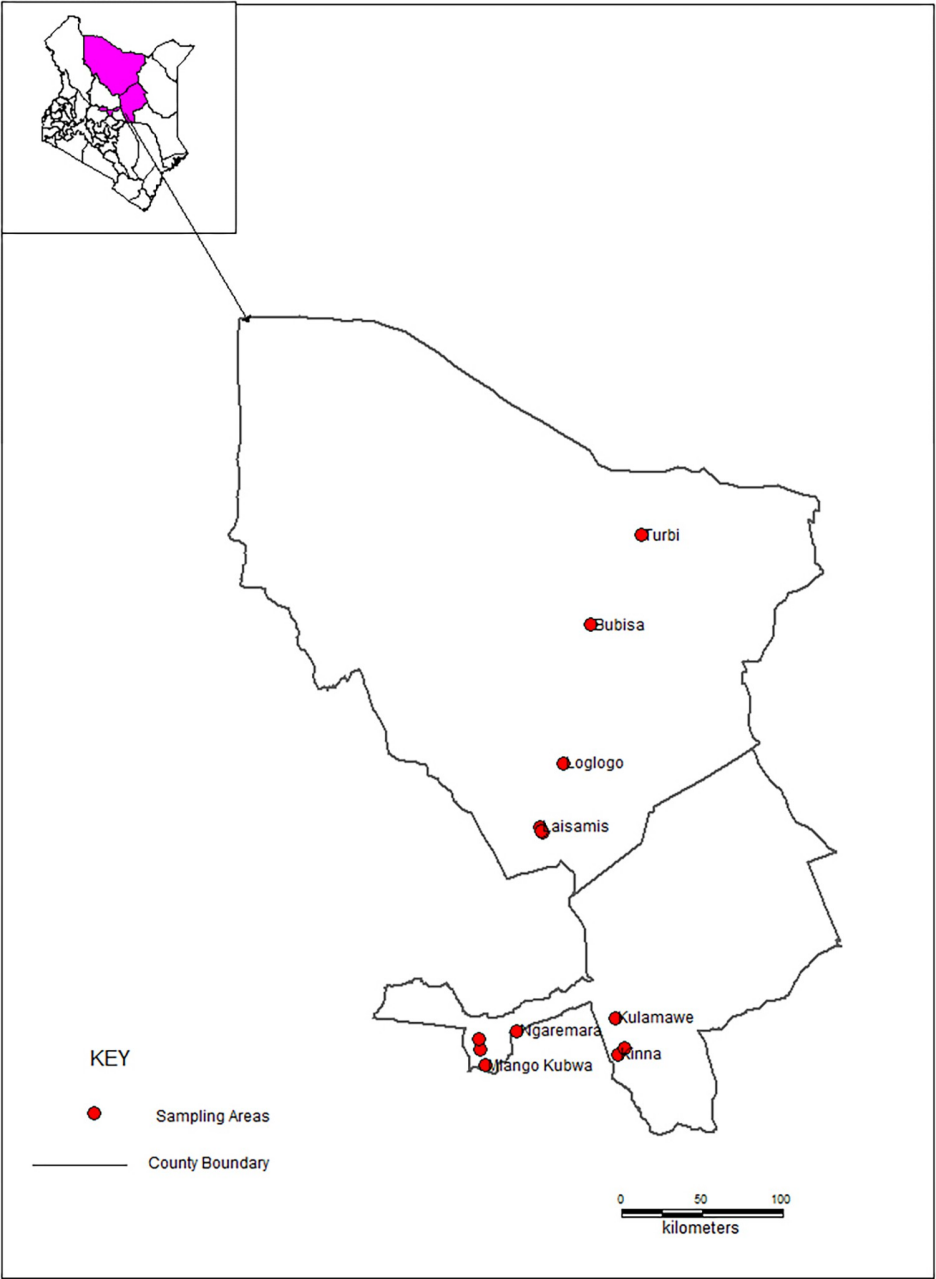
A map of Kenya showing the sampling sites in Isiolo and Marsabit counties (made with Natural Earth).

The study was conducted in Isiolo and Marsabit counties, Kenya. Isiolo County covers an area of about 25,349 square km land size. The county has a human population of 268,002, with a population density of about 11 persons/km^2^ according to the 2019 population and household census [51]. It is inhabited by four ethnic communities that include Meru, Borana, Somali and Turkana. Four study sites were selected in Isiolo namely Kulamawe (Borana-inhabited), Kinna (Borana-inhabited), Ngaremara (Turkana-inhabited) and the Somali-inhabited Livestock Marketing Division (LMD) based on ethnic composition of the camel owners and proximity to administrative centers as proxies to access to veterinary services. Marsabit County covers an area of about 70,944 square kilometers. The county borders the country of Ethiopia to the north. Marsabit is home to 459,785 people with a population density of 6 persons/km^2^ [51]. The county is inhabited by various ethnic communities including the Cushitic Rendille, Gabbra and Borana as well as the Nilotic Samburu and Turkana. Four study sites were selected namely Bubisa (Gabbra-inhabited), Turbi (Gabbra-inhabited), Logologo (Rendille-inhabited) and Laisamis (Rendille-inhabited) based on ethnic composition and proximity to administrative centers as proxies to access veterinary services. Camel production is an important source of livelihood for the ethnic communities in the present study. Map of Kenya showing the study sites in Isiolo and Marsabit counties is presented in **Fig 1.**

### Animal screening, collection and storage of samples

An epidemiological survey was carried out to screen for *Surra* in 8 preselected sites in Marsabit (Laisamis. Loglogo, Bubisa, Turbi) and Isiolo (Kulamawe, Ngaremara, Kinna, LMD) counties. A total of 2,750 camels from 160 herds belonging to different individuals were examined using buffy coat technique and Giemsa-stained dry smears for trypanosomes. The camels were examined at the beginning of dry season, at peak of dry season and during the wet season between September 2018 and April 2020. A total of 113 (4.1%) of the screened camels tested positive for *T*. *evansi* infection of which 100 isolates were used in the sensitivity study. Blood from all the camels infected with *T*. *evansi* were collected from the jugular vein into heparinized vacutainer tubes containing 20% glycerol and EDTA and cryopreserved in liquid nitrogen at -196°C as a stabilate.

### Infectivity of donor mice

One hundred (100) cryo-preserved *T*. *evansi* isolates were thawed in two batches of 60 and 40 respectively, and inoculated intraperitoneally (i.p) into outbred Swiss White adult (23–25 days old) female mice. Each stabilate was administered into two mice (24 hours after last dose of cyclophosphamide administered at 100 mg/kg via intraperitoneal route for 3 consecutive days as immunosuppressant) at a rate of 1 x 10^5^ trypanosomes/mouse via i.p route. The mice were monitored daily by collecting blood from the tail, expressing a drop on a microscope slide and examining it under dark field magnification X400 in a Leitz microscope. At peak parasitemia, blood from donor mice that developed an infection was collected and diluted appropriately with EDTA Buffer for inoculation into the experimental mice. Confirmation of trypanosome species was based on movement of the parasites under the microscope and morphological structures on dry stained smears as described by [1] (Giemsa-stained dry blood smears were prepared from all trypanosome isolates for trypanosome species identification). The dry smears were fixed with absolute methanol for 1 minute and stained with Giemsa (10%) for 45 minutes, dried and examined under oil immersion at X1000 magnification [52]. *T*. *vivax* was ruled out through mice inoculation as it rarely establishes infection in mice [53].

### Test drugs

Four trypanocides were tested at the recommended dosages: isometamidium chloride at 1mg/kg bwt, diminazene aceturate at 20mg/kg bwt, homidium bromide at 1mg/kg and quinapyramine sulphate & chloride at 1mg/kg bwt.

### Infectivity of experimental mice (In vivo mice sensitivity test)

In a randomized controlled trial (RCT) of a single-dose test as described by Eisler et al. [53], mice were randomly allocated into five treatment groups each containing 6 mice. Briefly; each mouse in all the treatment groups was injected with an inoculum containing *T*. *evansi* (0.2 ml of 5x10^4^ trypanosomes/ml) from the donor mice via intraperitoneal route. The mice were weighed before administration of treatments to an accuracy of 1 gram. All the body weights varied by less than 10%. A single differentiating dose for each drug was used to treat groups of mice 24 hours after inoculum injection at the following dosage rates:

**Table:** 

Treatment group	Treatment given	Dosage	Manufacturer	Batch no.
**Group 1**	Isometamidium chloride	1 mg/kg	Ceva Sante’ Animale France	TFP711A
**Group 2**	Diminazene aceturate	20 mg/kg	Ceva Sante’ Animale France	360A1 (lot)
**Group 3**	Homidium bromide	1 mg/kg	Laprovet France	
**Group 4**	Quinapyramine sulphate & chloride	1 mg/kg	Vetoquinol India animal Health Pvt. Ltd, India	STQ18008
**Group 5**	Distilled water (positive control)	0.2 ml	N/A	N/A

All the drugs were reconstituted so that the final drug dosage received by each mouse was contained in 0.2 ml of the final solution. After treatment, screening for trypanosomes in mice in all the treatment groups was done daily for the first 14 days and thereafter twice a week until day sixty (60) using dark ground/phase contrast buffy coat technique (magnification x250) of wet smear prepared from tail blood as described by Eisler et al. [53]. Results were recorded as negative or positive and scoring of parasitaemia carried out as described by Herbert and Lumsden [54]. Only experiments where at least 5 out of the 6 control mice became parasitaemic were considered valid [53]. Blood for parasitemia assessment was collected via tail nips which induces minimal pain thus does not require use of analgesia. There was no intrusive procedure conducted on the mice warranting use of analgesia or anesthesia. The mice were humanely euthanized when the parasitemia score remained at least 7.5 (Herbert and Lumsden score [54]) for five consecutive days through cervical dislocation. All the surviving mice were euthanized after day 60 through cervical dislocation.

### Interpretation of results

Trypanosome isolates were considered drug-sensitive if at least 5 out of the 6 treated mice got cured (remained aparasitaemic until the end of the 60-day observation period). If fewer than 5 mice were cured, the isolate was considered to express resistance to the dosage used. In case one test mouse died prior to detection of parasitaemia, the isolate was considered as drug-sensitive if at least 4 out of the remaining 5 mice were cured; if fewer than 4 were cured, the isolate was considered to express resistance to the dosage used. However, if more than one test mouse died prior to detection of parasitaemia, the test was either repeated or dropped [53].

### Survey of camel owners’ practices around *Surra* prevention, control and treatment

In order to explore the management practices among camel keeping ethnic communities in Marsabit and Isiolo counties that influence drug resistance, a cross-sectional survey was undertaken to 1) determine camel management practices across the communities, 2) evaluate the level of knowledge of *Surra* and its vectors across the communities and 3) identify specific measures applied by the communities in management of *Surra* and its vectors. The survey was undertaken in the same eight sites where animal screening was undertaken. A structured questionnaire administered by trained enumerators was used to capture relevant variables across the communities. A total of 340 respondents were interviewed distributed as follows: Somali (111) from LMD, Rendille (69) from Laisamis and Logologo, Gabbra (84) from Bubisa and Turbi, Borana (35) from Kulamawe and Kinna, and Turkana (41) from Ngaremara. The questionnaire captured the following aspects:

- **Camel husbandry practices of different communities.** The respondents were asked to respond to husbandry related questions focusing on pasturing practices. The questions asked whether the farmers avoided infested areas during grazing and if they applied insecticides on camels among other aspects. The complete questionnaire is given as **S1 Appendix**.
- **Knowledge of camel trypanosomiasis, vectors and its management.** Knowledge of *Surra* was assessed by evaluating camel owners’ awareness of the main clinical signs as explained in Desquesnes et al. [10], (see **S2 Appendix** for the list of clinical signs assessed). However, since these signs are not unique to *Surra*, but can manifest in other camel diseases present in the area [55], herders were required to give possible source of the clinical signs by describing the vectors as well as the options available for treatment of the symptoms. Clinical signs used were the 12 symptoms listed by Kula et al. [56]. The ability of the farmers to mention six clinical signs was arbitrarily selected as cut off point in the computation of knowledge level so as to get an understanding of herders’ knowledge of symptoms associated with both acute and chronic forms of *Surra*. Vector awareness was guided by herders’ pictorial identification of any of the vectors listed by Desquesnes et al. [1], including description of their habitat and seasonality. Assessment of *Surra* treatment knowledge entailed camel owners’ correct description of use of any of the drugs listed in Giordani et al. [21]. Herders were scored on each of the three areas and those adjudged to have good knowledge of *Surra* had to describe at least six correct clinical signs, identify at least one vector and describe proper use of at least one therapeutic or prophylactic drug.
- **Management of *Surra* and its vectors by the different communities.** In assessing *Surra* management options used by different communities, respondents were required to state the time (date and month) the last case of *Surra* occurred in their herds; whether the case was treated, who undertook the treatment, approach used to treat the last case; name of product used in treating the case, how the product was prepared during the treatment, if multiple trypanocides were used and treatment outcome (**S1 Appendix**). The drug sensitivity results were interpreted together with epidemiological prevalence data and socioeconomic data on trypanocidal drug use, insecticidal use, level of education, herd dynamics and use of ethno-veterinary products in the study sites.

### Data analysis

The data from mice sensitivity tests, prevalence and survey of camel owners’ practices were collated and inputted in Microsoft Excel® sheet and imported into STATA version 14 for analysis of frequencies, descriptive statistics, and association tests. The descriptive statistics were done and presented as mean ± standard deviation (mean ±SD). The prevalence of drug resistance in each study site was determined as a percentage of the number of isolates considered to be resistant to a particular drug (as per interpretation of mice results above) over the number of isolates tested from that study site. The prevalence of *T*. *evansi* was the number of camels that tested positive for *T*. *evansi* over the total number of camels sampled in the study sites. A one way and two-way ANOVA was conducted to determine mean differences in age of household heads, years of formal education and average household size by community orientation. Multivariate regression model was used to determine the effect of drug use on drug resistance and trypanosomiasis prevalence and the effect of insecticide use, use of ethno veterinary products and treatment of camels by the pastoralists on the prevalence of *T*. *evansi* infection in camels. Socioeconomic data on use of trypanocidal drugs and insecticides, level of education, herd dynamics and use of ethno-veterinary products in the study sites were converted to proportion (%) of respondents in each study site who answered ‘Yes’ to the questions relating to the various variables considered as independent variable in the multivariate regression analysis while the prevalence of *T*. *evansi* (%) in each study site was considered as the dependent variable. All significant levels were stated at p<0.05.

## Results

### Demographic characteristics of the interviewed camel keeping households

A total of 340 household heads comprising of 83.5% men and 16.5% women were interviewed in the study. Distribution of respondents by ethnic community was as follows: Somali (111), Turkana (41), Borana (46), Gabbra (83) and Rendille (72). The main source of income for majority of the households was livestock production (63.5%), followed by business (13.6%), formal employment (2.9%), while 2.4% of the households practiced crop farming. Up to 17.6% of respondents derived their livelihoods from other sources such as informal employment and pension. Disaggregation of the camel farmers by ethnic community, years of formal education, size of household and age of household head is presented in **Table 1**.

**Table 1:** Disaggregation of the camel farmers by ethnic community, years of formal education, size of household and age of household head.

Demographic characteristic	Ethnic community					
	Somali (N = 111)	Rendille (N = 72)	Gabbra (N = 83)	Borana (N = 46)	Turkana (N = 41)	Total (N = 340)
Household head with formal education (n)	73	67	72	22	34	268
Years of formal education of household head (mean ±SD)	2.59±4.0^a^	0.32±2.0	1.1±3.5	2.7±4.6 ^a^	1.3±3.2	1.6±3.6
Age of household head (years) (mean ± SD)	54.8±15.1	52.9±12.3	55.5±14.8	47.6±15.5	46.6±15.6	52.9±14.9
Size of household (number) (mean ± SD)	9.9±4.4	10.0±5.5	7.6±2.7	9.3±3.7	10.5±5.1	9.4±4.4

Values across rows with the same superscript were significantly different at p<0.05.

## Practices of the camel keepers around prevention, control and treatment of *Surra*

**Fig 2:**
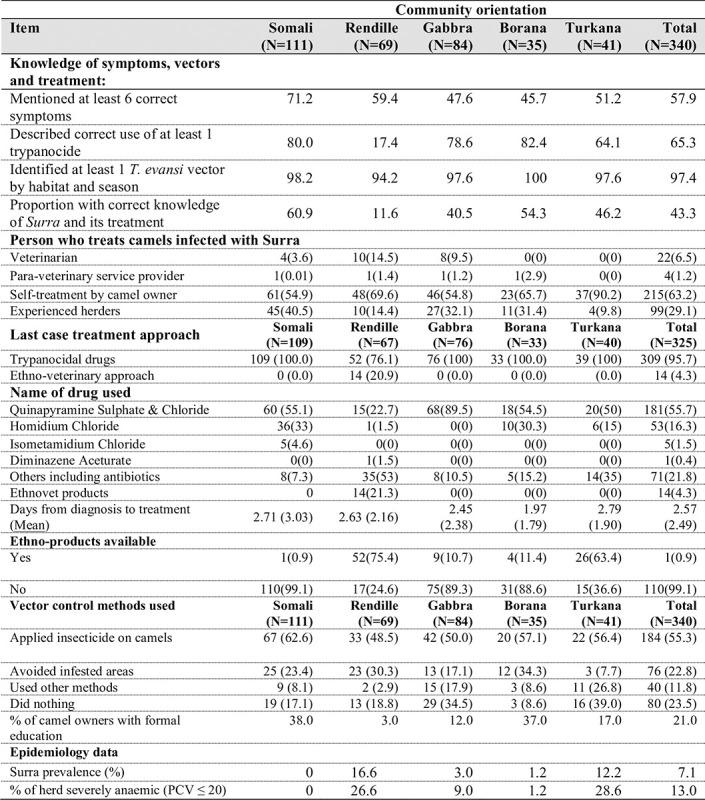
Characterization of community knowledge and practices around camel *Surra*.

Data from camel owners’ practices revealed that knowledge of *Surra* was highest among Somali and lowest within Rendille ethnic communities, being significantly different across the 5 communities (*p*<0.00; χ2 = 44.26; df = 4). There were no significant differences across communities in the proportion of herders who were able to mention at least six correct clinical signs of the disease. Majority of the camel keepers carried out self-treatment (63.2%) of *Surra* infected camels whereas 29.1% and 7.7% procured services of experienced herders and veterinary professionals, respectively, in treating the sick camels. A high proportion of the camel keepers used trypanocidals (95.7%) to treat *Surra* while 4.3% used herbal remedies. Quinapyramine sulphate & chloride was the most commonly used trypanocide (55.7%) followed by homidium chloride (16.3%). Detailed characterization of community knowledge and practices around *Surra* is given in **Fig 2**.

### Sensitivity of the isolates to trypanocides

A total of nineteen (19) of the Isolates tested were collected from Marsabit County (Laisamis, Loglogo, Bubisa and Turbi) and 21 from Isiolo County (Ngaremara, Kulamawe and Kinna). All camels screened at LMD sampling site in Isiolo tested negative for *T*. *evansi* and therefore there was no isolate from this site. **Table 2** shows the distribution of tested isolates by location and sensitivity results.

**Table 2:** Number of tested isolates that were sensitive or resistant to various drugs used for *Surra* treatmet in Marsabit and Isiolo counties.

County	Sampling site	Sensitivity	HOM	ISMM	QPY	DIM
Isiolo	Kinna	Resistant	2/2	2/2	2/2	0/2
		Sensitive	0/2	0/2	0/2	2/2
	Ngaremara	Resistant	9/10	6/10	8/10	0/10
		Sensitive	1/10	4/10	2/10	10/10
	Kulamawe	Resistant	8/9	8/9	8/9	2/9
		Sensitive	1/9	1/9	1/9	8/9
Marsabit	Laisamis	Resistant	8/10	6/10	8/10	2/10
		Sensitive	2/10	4/10	2/10	8/10
	Turbi	Resistant	4/6	2/6	3/6	1/6
		Sensitive	2/6	4/6	3/6	5/6
	Logologo	Resistant	½	½	½	0/2
		Sensitive	½	½	½	2/2
	Bubisa	Resistant	1/1	1/1	1/1	0/1
		Sensitive	0/1	0/1	0/1	1/1

**Numerator** = number of isolates either sensitive or resistant to the drug.

**Denominator** = total number of isolates tested from the location.

HOM = Homidium chloride/bromide.

ISMM = Isometamidium Chloride.

DIM = Diminazene aceturate.

QPY = Quinapyramine sulphate & chloride.

Of the 40 isolates tested, 33(82.5%) were resistant to the trypanocidal drugs used in the study site. Isolates resistance to all the four drugs tested were observed in 2 (Kulamawe and Laisamis) of the 7 sites sampled and isolates sensitive to all the 4 drugs tested were observed in 5 of the 7 sites sampled. Five (12.5%) of the 40 isolates tested were resistant to diminazene aceturate whereas, 25(62.5%), 30(75%) and 31(77.5%) were resistant to isometamidium chloride, quinapyramine sulphate & chloride and homidium chloride, respectively. Seven (17.5%) isolates of the 40 tested were sensitive to all 4 drugs, whereas, 7.5%, 10%, 55% and 10% were resistant to 1,2,3 and 4 drugs respectively. Therefore, the overall prevalence of multiple drug resistance (resistance of an isolate to more than one drug) in the two counties was 75.0% as presented in **Table 3**.

**Table 3:** The number of tested isolates from study sites in Marsabit and Isiolo that were sensitive or resistant to one, two, three or all the four drugs tested.

County	Sites	Level of resistance	Number of isolates (n)	Percentage of isolates (%)
Isiolo	Kinna	S1/R3	2	100
	Ngaremara	S1/R3	6	60
		S2/R2	1	10
		S3/R1	2	20
		S4	1	10
Marsabit	Laisamis	R4	2	20
		S1/R3	4	40
		S2/R2	2	20
		S4	2	20
	Loglogo	S1/R3	1	50
		S4	1	50
	Bubisa	S1/R3	1	100
	Turbi	S1/R3	2	20
		S2/R2	1	20
		S3/R1	1	20
		S4	2	40

S = Sensitive, R = Resistant, S4 = Sensitive to 4 drugs, S3/R1 = Resistant to 1 drug; S2/R2 = Resistant to 2 drugs; S1/R3 = resistant to 3 drugs; R4 = Resistant to all 4 drugs.

Of the 21 isolates from Isiolo County tested 19(90.5%) were resistant to the trypanocidal drugs used in the study counties. Two (9.5%) isolates were resistant and 2 were sensitive to all the four drugs tested. Whereas, 2 (9.5%), 16 (76.2%), 18 (85.7%) and 18(85.7%) isolates were resistant to diminazene aceturate, isometamidium chloride, quinapyramine sulphate & chloride and homidium chloride, respectively (**Table 2**). Fourteen (66.7%), 1(4.8%) and 2(9.5%) were resistant to 3, 2 and 1 drug, respectively (**Table 3**). Consequently, the prevalence of multiple drug resistance in Isiolo was 81.0%. Prevalence of resistance against diminazene aceturate was 22.2%, whereas resistance against isometamidium chloride, homidium and quinapyramine sulphate & chloride varied from 60% to 100%. **Tables 4 and 5** presents summarized resistance results from the different sampling sites.

**Table 4:** Number of isolates resistant to a drug per site.

County	Site	HOM	ISM	QPY	DIM
**Isiolo**	Kinna	2 (100%)	2 (100%)	2 (100%)	0
	Ngaremara	8 (80%)	6 (60%)	8 (80%)	0
	Kulamawe	8 (88.9%)	8 (88.9%)	8 (88.9%)	2 (22.2%)
**Prevalence**		85.70%	76.20%	85.70%	9.50%
**Marsabit**	Laisamis	8 (80%)	6 (60%)	8 (80%)	2 (20%)
	Turbi	4 (66.7%)	2 (33.3%)	3 (50%)	1 (16.7%)
	Loglogo	1 (50%)	1 (50%)	1 (50%)	0
	Bubisa	1 (100%)	1 (100%)	1 (100%)	0
**Prevalence**		73.70%	52.60%	68.40%	15.80%
**Overall prevalence**		80.0%	65.0%	77.5%	12.5%

HOM = Homidium chloride.

ISM = Isometamidium Chloride.

DIM = Diminazene aceturate.

QPY = Quinapyramine sulphate & chloride.

**Table 5:** Prevalence of trypanocidal drug resistance in the study sites in Marsabit and Isiolo counties.

County	Sites	Isolates resistant to drugs	Prevalence of drug resistance
**Isiolo**	Kinna	2	100%
	Ngaremara	9	90%
	Kulamawe	8	88.9%
**Marsabit**	Laisamis	8	80.0%
	Loglogo	1	50.0%
	Bubisa	1	100.0%
	Turbi	4	66.7%

Within the three sites in Isiolo county prevalence of trypanocidal resistance varied from 88.9% in Kulamawe to 100% in Kinna (Table 5). One isolate in Ngaremara and Kulamawe respectively was sensitive to all the four drugs tested, while 2 (22.2%) isolates in Kulamawe were resistant to all the drugs tested. Therefore, the prevalence of multiple drug resistance was 100% in Kinna, 70% in Ngaremara and 88.9% in Kulamawe (Table 3).

Of the isolates from Marsabit County tested, 73.7% were resistant to the trypanocidal drugs used in the study counties. Two (10.5%) Isolates were resistant while 5 (26.3%) were sensitive to all four drugs tested. Three (15.8%), 10 (52.6%), 13 (68.4%) and 14 (73.7%) isolates were resistant to diminazene aceturate, isometamidium chloride, quinapyramine sulphate/chloride and homidium respectively (Table 2), whereas 8(42.1%), 3(15.8%) and 1(5.3%) were resistant to 3, 2 and 1 drug respectively (Table 3). Consequently, the prevalence of multiple drug resistance in Marsabit was 73.7%. Within the 4 sites in Marsabit County, prevalence of trypanocidal resistance varied from 50.0% in Loglogo to 100.0% in Bubisa (Table 5). Prevalence of resistance against diminazene aceturate varied from a minimum of 16.7% resistance (in Turbi), to a maximum of 20% (in Laisamis). Prevalence of resistance against ISM, HOM and QPY varied between 33.3% to 100%, 66.7% to 100% and 50% to 100% for ISM, HOM and QPY respectively (Table 4). Two (22.2%), 1 (50%) and 2 (40%) isolates in Laisamis, Loglogo and Turbi respectively were sensitive to all the 4 drugs tested, while 2 (20%) isolates in Laisamis were resistant to all the drugs tested. Therefore, the prevalence of multiple drug resistance was 80% in Laisamis, 50% in Loglogo, 100% in Bubisa and 60% in Turbi (Table 3).

**Fig 3:**
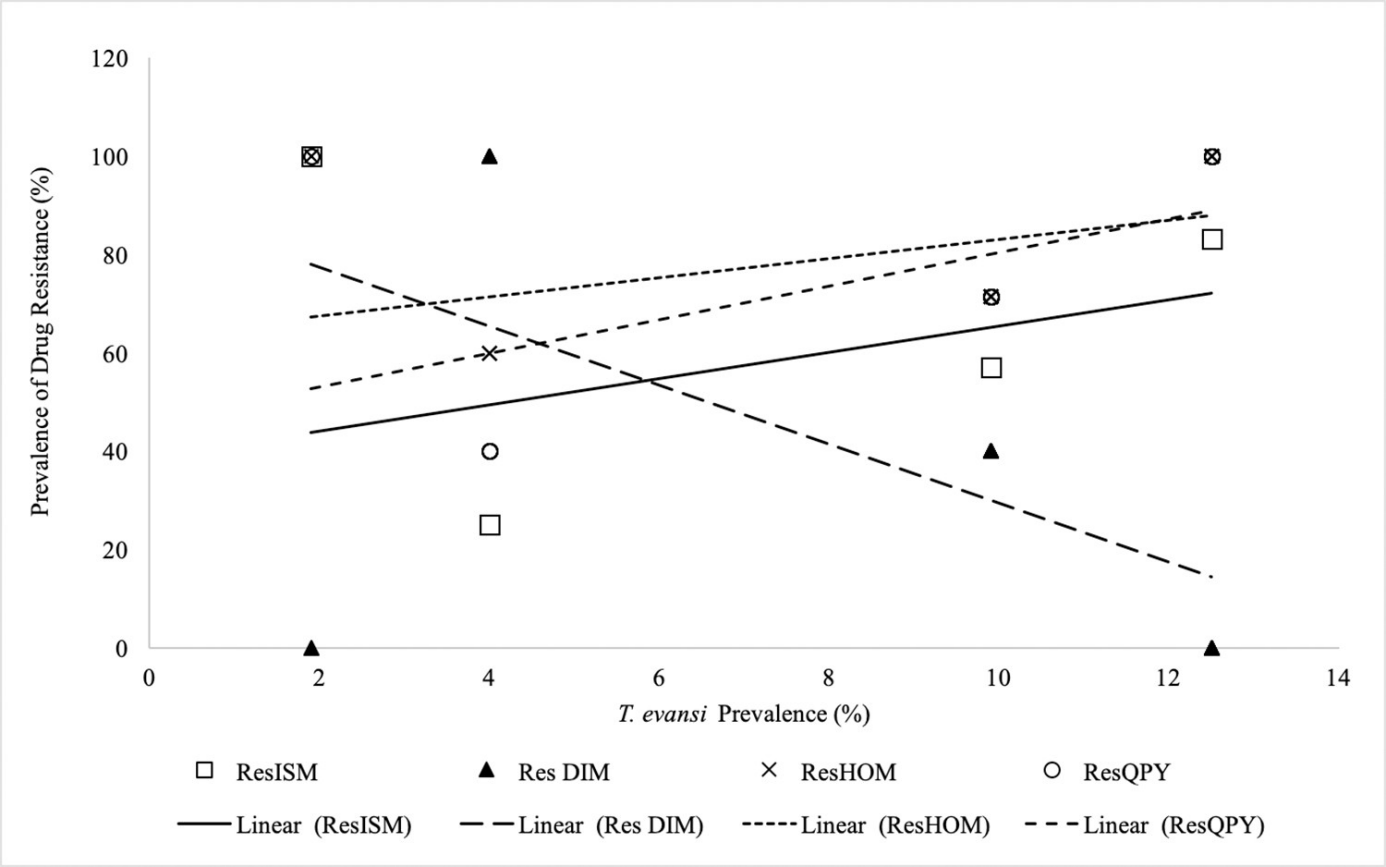
Variation of various drug resistance with prevalence of *T*. *evansi* in Isiolo and Marsabit counties.

The prevalence of camel *Surra* in the study area affected the prevalence of isolates resistant to various drugs differently (**Fig 3**).

The prevalence of isolates resistant to HOM (p = 0.049), ISM (p = 0.073) and QPY (p = 0.010) increased with prevalence of *T*. *evansi* infections. However, prevalence of isolates resistant to DIM decreased significantly (P = 0.006) with increase of prevalence of *T*. *evansi* infections. Trypanocidal drug use by the different ethnic communities affected the prevalence and the degree of drug resistance isolates in the counties. The increase in HOM, QPY and DIM use in the counties significantly (p = 00001) increased the prevalence of HOM, QPY and DIM resistance, respectively. However, use of isometamidium by pastoralists had no effect (p = 0.888) on the prevalence of isometamidium drug resistance in the study area. Mixing of trypanocidal drugs with other drugs or use of ethno-veterinary compounds had no effect (p = 0.314) on the prevalence of drug resistance. Increase in insecticidal use by pastoralists in the study area increased (p = 0.0001) the prevalence of drug resistant isolates. The proportion of pastoralist with no formal education in an area had a significant (p = 0.013) effect on the prevalence of drug resistance of isolates from the area. The higher the lack of formal education among the pastoralist the lower the prevalence of drug resistant isolates recorded. In contrast, the proportion of pastoralists with formal education had no significant effect on the prevalence of drug resistant isolates in an area. Herd dynamics influenced by inflows such as new births, purchases, gifts exchanges had no effect (p = 0.445) on the prevalence of drug resistance in the study area.

**Fig 4:**
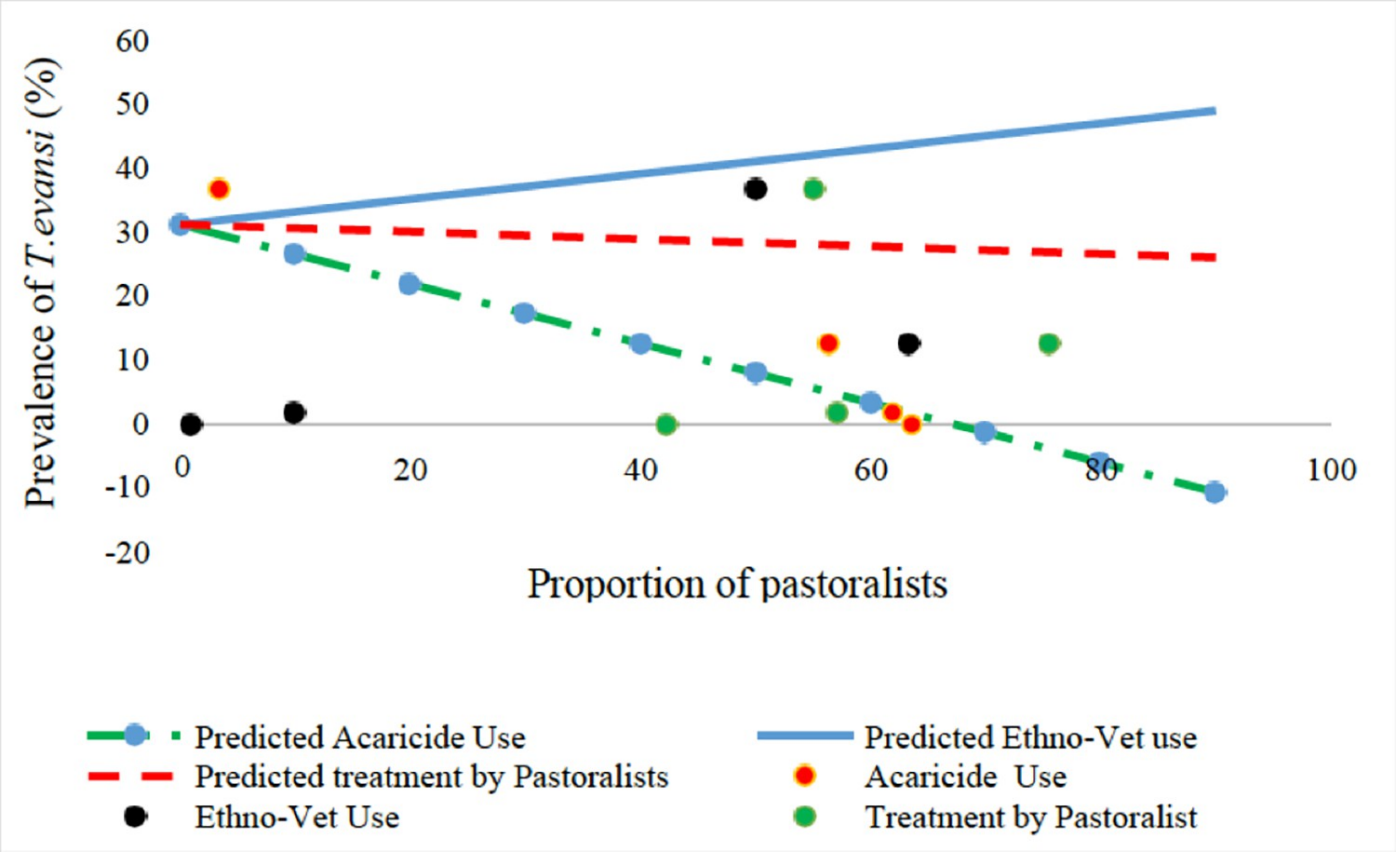
The effect of insecticide use, ethno veterinary compound use and treatment of camels by the pastoralists on the prevalence of *T*. *evansi* infection in camels in Isiolo and Marsabit counties.

The herd prevalence of *T*. *evansi* in camels decreased with the increase in insecticidal use (p = 0.065), homidium use (p = 0.005), avoiding of suspected high fly density areas (p-0.082) and increase in female to male ratio of camels in the study area. In contrast, the herd prevalence of *T*. *evansi* increased significantly (p = 0.007) with the increase in the use of Ethnoveterinary products (**Fig 4**) and increase in the knowledge of clinical signs of *Surra*. In contrast, the proportion of pastoralist treating their own camels had no significant (p = 0.405) effect on the prevalence of *T*. *evansi* in camels despite frequent treatment (52%) by the pastoralists (**Fig 4**).

## Discussion

The overall prevalence of drug resistance was 82.5% indicating the circulating *T*. *evansi* populations exhibit reduced sensitivity (resistance) to the available trypanocidal drugs in the two counties. However, this also indicates there are still *T*. *evansi* populations circulating that would respond to treatment if administered correctly. Instability of drug resistance in bacteria and trypanosome populations has been observed in response to withdrawal of antibiotic or trypanocidal due to selective pressure [57]. This implies that the observed drug resistance in the two counties is not permanent and may vary with time due to inconsistency of the selective drug pressure which is dependent on availability of the drugs to the pastoralist communities and their usage. From a similar sensitivity study carried out more than six years earlier in the two study counties, the prevalence of resistance to diminazene, isometamidium and quinapyramine decreased following withdrawals of the drugs [15]. Resistance by field isolates to ISM, HOM, DIM, quinapyrammine have been previously demonstrated in other studies [34,58–62]. It is imperative then that trypanocidal drugs should only be used upon accurate disease diagnosis so as to reduce drug pressure on the trypanosome populations. However, majority of the camel owners in the present study carried out self-treatment of sick camels instead of using qualified veterinarians, a practice that may be contributing to development of drug resistance. Self-treatment of sick camels by unqualified farmers has previously been associated with wrong route of administration, wrong reconstitution and wrong dosages all of which may lead to development of drug resistance as has been reported for diminazene aceturate and Isometadidium chloride [40].

Despite the resistant isolates being observed in all the seven sites where trypanosome isolates were collected, only two sites had circulating trypanosome populations that were resistant to all the four drugs being used in the region, whereas five of the seven sites had circulating trypanosome populations that were sensitive to all the drugs used in the region. This is an indication that control of *Surra* using drugs would still be effective in the region if used properly according to the manufactures prescription. Previous studies showed that increasing dosage and repeat application of some trypanocidal drugs can be effective in eliminating the infection as long as the therapeutic index is observed [58–60,63]. A single dose test was used in the present study and it may be interesting to know how the resistant isolates may respond to increased trypanocidal doses. The level of intensity of trypanocidal drug use by the communities was associated with increase in prevalence of drug resistance especially for the drugs that were in frequent use (QPY, ISM and HOM). The intensive use of trypanocidal drugs for the control of trypanosomiasis has been associated with appearance of drug resistant trypanosomes [33,64,65]. This suggests that rational use of trypanocides where treatment is administered correctly (right dose and route of administration) in animals definitively diagnosed to be trypanosome infected would reduce the incidence of drug resistance.

Despite ready availability of diminazene aceturate in the two counties, majority of the isolates (90%) were sensitive to this drug. This may be attributed to the limited use of this drug in treatment of *Surra* by pastoralists in the two counties relative to other trypanocides as our survey revealed. diminazene aceturate in powder formulation has been observed to be toxic to camels at 10mg/kg and therefore not recommended for use in camels [66]. Also, diminazene aceturate is rapidly eliminated from the system after administration which minimizes development of drug resistance compared to other more persistent trypanocidals [67,68]. However, a liquid formulation of diminazene aceturate with phenazone and procaine hydrochloride has been recommended for *Surra* treatment in camels [69,70] and is available in the study counties, although its efficacy in treatment of *T*. *evansi* infections is questionable [71].

The 12% isolates that exhibited resistance to diminazene aceturate were collected from Marsabit county in areas inhabited by the Rendille community whose knowledge of use of drugs was limited and only used quinapyramine sulphate & chloride in treatment of *Surra*. It is likely that the observed diminazene aceturate resistance is as a result of cross resistance from quinapyramine sulphate & chloride. Early studies by whiteside [38,72] concluded that induction of resistance to quinapyramine under field conditions appeared to result in cross-resistance to homidium chloride, isometamidium chloride and diminazene aceturate. In the present study, a significant difference in the prevalence of multiple drug resistance between isolates from the two counties was observed which may be attributed to differences in trypanocidal drug usage between pastoralist communities in the two counties. In a previous study, a high proportion of pastoralist in isiolo used homidium chloride and quinapyramine sulphate & chloride respectively whereas the majority of pastoralist in Marsabit county used either antibiotics or herbal concoctions as an alternative treatment of what they perceived to be *Surra* [26].

Extensive use of trypanocidal drugs and under dosing of drugs has been indicated as the main contributor to development of drug resistance due to exposure of trypanosomes to sub-therapeutic drug levels, thereby favoring the selection of drug-resistant populations [33]. The presence of multiple drug resistant *T*. *evansi* populations circulating in the two study counties is of concern. However, the fact that these populations are sensitive to diminazene aceturate suggests that use of ‘sanative pair’ of drugs may still be applicable in the control of *Surra* in Marsabit and Isiolo. The ‘Sanative pair’ are pairs of drugs that do not induce resistance to each other and can be used alternatively in the field when resistance to either drug appears [36,72]. Whiteside [72] proposed that homidium and diminazene or isometamidium and diminazene should be used together as ‘sanative’ pairs of drugs in areas where drug resistance has been observed. In addition, the multiple drug resistance observed in this study is at the population level and most likely not at the individual trypanosome level where treatment using a “sanative pair” would have been impossible [42]. Ndungu et al. [30] demonstrated that several clones derived from an isolate that exhibited resistance to trypanocidal drugs in mice showed variable sensitivity to treatment. Whether the clones from the multiple resistant isolates from this study could have variable sensitivity to diminazene, homidium and isometamidium was not investigated.

However, the observation in the present study that trypanosome populations circulating in the study region that demonstrated resistance to the four drugs tested were located in only two of the seven sites further suggests multiple drug resistance is not widely spread in the region at present. Therefore, control of *Surra* using drugs would still be effective if used rationally as per label prescription. It is well known that–although there is a quite good correlation of the mouse test with the test in ruminants–the curative dose in mice cannot be extrapolated to livestock [19]. However, the standard mouse test (used in this study) has been used effectively as a single dose test for epidemiological purposes in studying of area-wide occurrence of drug resistance in various regions of Africa [73] allowing spatial and temporal comparisons or as a multi-dose test, which measures the degree of resistance in individual trypanosome isolates [53]. The present study established high drug resistance levels in areas with high *T*. *evansi* prevalence. The association between prevalence and resistance to trypanocides has been recorded elsewhere [61]. High prevalence of treatment failure of trypanocides in cattle that was attributed to drug resistance has been observed in seasons of high tsetse and trypanosomosis challenge in the coastal areas of Kenya [32]. Since the herd prevalence decreased with use of insecticides, avoiding vector infested areas and *Surra* knowledge, these practices should be integrated in *Surra* control strategies. The relatively lower prevalence recorded in herds with more female camels can be attributed to the comparatively better management accorded to female camels by pastoral communities due their economic importance [74].

In conclusion, the multiple drug resistance observed in the two counties is not an indication of total trypanocidal drug treatment failure. Use of “sanative pair” of drugs as the commonly accepted method for delaying the development of drug resistance may most likely be still applicable in the region since there were a number of isolates that were resistant (10%) to a single drug and sensitive (17.5%) to all the drugs from five out of the seven sites sampled. In addition, judicious treatment of confirmed cases of *Surra* with adequate amount of drug would still be able to effectively control the disease in the region especially since the observed resistance appears to be at the population level and not at the clonal (individual trypanosome) level. However, cloning and the individual characterization of trypanosomes from the multiple drug resistant isolates may be necessary to clarify whether or not the sanative pair can still be used. Trypanosome isolates collected from the field as was the case in the present study have been shown to consist of a panmictic population of trypanosomes resistant to a single drug or a homogenous population of multi-resistant trypanosomes or a mixture of both together. In each of these situations different measures will have to be taken to avoid further worsening of the situation. To achieve that, further molecular characterization is required. A moderately high trypanosomiasis prevalence was recorded using buffy coat technique and Giemsa-stained dry smears despite their moderate sensitivities implying the prevalence could be even higher. Consequently, strategic use of integrated control of the disease and the vector using available alternative methods to reduce drug use is necessary to avoid rapid development of drug resistance. Integrated approaches have been shown to lead to reduced disease prevalence under reduced drug use. Such a strategy would comprise of (i) judicious use of trypanocides in definitively diagnosed tsetse positive animals, (ii) control of vectors by targeted bi-monthly insecticidal spraying of the animals, (iii) strategic deworming in the beginning and the end of the rainy season and, (iv) strategic use of prophylaxis when grazing through highly vector infested areas.

## Supporting information

S1 Appendix Questionnaire on camel owners’ practices around *Surra* prevention, control and treatment. (PDF)

S2 Appendix Clinical signs the camel herders associated with camel *Surra*. (PDF)
