# Supplementary material for: Variation of sensitivity of Trypanosoma evansi isolates from Isiolo and Marsabit counties of Kenya to locally available trypanocidal drugs
Source: PLoS One. 2023 Feb 2;18(2):e0281180. doi: 10.1371/journal.pone.0281180 (PMC9894490; doi:10.1371/journal.pone.0281180)
Supplement: S1 Appendix — (PDF) [file pone.0281180.s001.pdf]

Assigned household number [_____________]

Intensifying Camel Productivity through Integrated Control of *Surra* within the Somali ecosystem of the Horn of Africa

Questionnaire Identification

ID1. Date of interview (dd/mm/yy)	___ ___/___ ___/ 2018
ID2. Settlement/Village	1. Gambella<br>2. Kina/Kulamawe<br>3. Laisamis<br>4. LMD/Mlango<br>5. Loglogo<br>6. Ngaremara<br>7. Turbi/Bubisa<br>8. Oldonyiro<br>[_______________]
ID3. GPS readings	N ______________________________<br>E ______________________________
ID4. Sub-County	1. Isiolo<br>2. Garbatula<br>3. Merti<br>4. North Horr<br>5. Maikona<br>6. Laisamis<br>[_______________]
ID5. County	1. Isiolo<br>2. Marsabit
ID6. Name of interviewer	____
ID7. Contact address for interviewer	Tel. No. ____________________________________<br>email address________________________________
ID8. Name of Respondent and Ethnic group	1. Name [__________________________]<br>2. Ethnic group [_______________]
ID9. Gender of Respondent	1. Male<br>2 Female [______________________]
ID10. Relationship of respondent to household head	1. Household head<br>2. Spouse<br>3. Son<br>4. Daughter<br>5. Other (Specify...<br>[______________________]
ID11. Filled questionnaire checked by?	

A. Personal profile of the camel producer

A.1 Name of household head ___________________________________

A.2 Gender of household head: 1. Male [_______] 2. Female [_______]

A.3 Age of household head [________] (years)

A.4 Years of formal education of household head [_________________]

A.5 Main occupation of the household head [_________________]

1= Livestock keeping 2= Formal employment 3=informal employment 4= Business 5= Student

6= (specify)................................

A.6 When did the household head start keeping camels? [_________] (Year)

A.7 (a) What is the form of ownership of the land you mainly use for livestock rearing? [_________]

1= Privately owned 2= Rented 3= Communally owned 4= Other forms of ownership (specify)..............................

(b). If land is privately owned or rented, what is the number of hectares?

Rented [____________] hectares Private [____________] hectares

B. Information on herd of camels kept and ownership

B.1 Please indicate the current number of camels in your herd and who they belong to?

**Type**	Number	Owner (Indicate in order of importance based on proportion owned)
B.1.1 Calves (less than a year)		[______][______][______] [______](codes)
B.1.2 Young female (pre-breeding)		[______][______][______] [______](codes)
B.1.2 Young male (pre-breeding)		[______][______][______] [______](codes)
B.1.3 Adult female (breeding age)		[______][______][______] [______](codes)
Lactating females		[______][______][______] [______](codes)
B.1.4 Males (entire)		[______][______][______] [______](codes)
B.1.5 Males (castrated)		[______][______][______] [______](codes)
B.1.6 Total		[______][______][______] [______](codes)

Codes

**Owner**	5=Female child
1=Household head<br>2=Spouse<br>3=Jointly by household head and spouse<br>4=Male child	6= Relatives<br>7= Neighbours<br>8=Business partners<br>9=Others (specify) ______________________

C Camel husbandry practices

C.1 What type of production systems do you practice? [__________________]

1= Agro-pastoralism (settled with cropping activities and movement of livestock out of homestead for several days)
2= Mixed crop/livestock (permanently settled, animals move out and return to homestead every day)
3= Semi Nomadic (settled with movement of livestock only over several days)
4= Fully –nomadic (household moves with animals)
5= Others (specify..

C.2 Who plays the following roles or makes the following decisions about camel production in your household?

**Decision/Role**	**<u>List 3 most important in order of importance</u>**<br>1= The Household head<br>2= The spouse in Household<br>3= Jointly in Household<br>4= Male children in Household<br>5= Female children in Household<br>6= Employed herder in household<br>7= Jointly with others (fora)<br>8= Other community members – specify
Who mainly contributes the labour for grazing the camels?	[______][______][______]codes
Who contributes labour to milking the lactating camels?	[______][______][______]codes
During milking, who makes the decision on the number of quarters to milk?	[______][______][______]codes
Who decides about sales or purchases of your camels?	[______][______][______]codes
When your camel falls sick who decides;<br>i) Whether to treat the camel?<br>ii) The type of treatment to give (conventional or traditional)<br>iii) Type of drugs to buy	 [______][______][______]codes<br>[______][______][______]codes<br>[______][______][______]codes
Who treats sick camels in your herd?	[______][______][______]codes
Who decides where to graze the camels?	[______][______][______]codes
Who decides when to migrate to new grazing areas?	[______][______][______]codes
<u>List other important roles</u>, Specify:	[______][______][______]codes
	[______][______][______]codes
	[______][______][______]codes

C.3 (i). Do you provide supplementary feeds to your camels apart from grazing? [____________]

1. Yes[________] 2. No[________]

(i) If YES above, what are these supplementary feeds – ***insert codes for all*** [_______][_______][_______]

1= Pastures
2= Fodder
3= Mineral salts
4= Feeds
5= others, specify ____

(ii) From where do you get the supplementary feeds? (***give name(s)***

____

____

____

C.4.(i) On average, on a good day, how much milk in litres do you milk from your herd?

[______________ litres/day]

(ii) How many litres of milk does your best camel produce per milking session?

[______________ litres]

(iii) More often than not, how many **quarters** of the **camel's udder** do you milk? [_____________]

(iv) More often than not, how many times do you milk your camels in a day? [_________]times/day

(v) Do you sell milk you get from your camel herd? 1. Yes[________] 2. No [________]

(vi) (a) If **YES above**, how many litres of milk do you sell? [________/litre(s)]

(b) What is the price per litre? [Kshs_____________/litre]

(vi) Where do you sell your milk?

1. Neighbours, 2. Brokers/middlemen 3. Market 4. Dairy 5. Others, specify……………….

(vii) If milk is sold, how far is the selling point from your household?

Name of place [___________________________];

Distance [_____________km]

C.5. Apart from rearing camels, what other livestock species do you keep and which household member actually owns the animals?

Animal species	Number of animals kept	Who owns the animals?
B.4.a Cattle	[_________]	[_________](code)
B.4.b sheep & goats	[_________]	[_________](code)
B.4.c Donkeys	[_________]	[_________](code)
B.4.d Chicken	[_________]	[_________](code)
B.4.e Others (specify)____________	[_________]	[_________](code)

Codes

**Owner**	
1=Male household head<br>2=Female household head<br>3=Male household head and his spouse<br>4=Female spouse<br>5=Male child	6=Female child<br>7=Relatives<br>8= Neighbours<br>9=Business partners<br>10=Others (specify) __________________________

C.6. (i) Do you graze your camels separately from your other livestock 1. Yes[________] 2. No[________]

If **YES**, why do you separate your camels from other species of livestock? List reasons below:

____

____

____

(ii) If **No**, which other livestock do you graze alongside your camels? [_______][________][________]

1=Goats 2=Sheep 3=Cattle 4=Donkeys

C.7. (i) Do you split your camels into different herds? 1. Yes[_______] 2. No[_______]

What is the basis of splitting the herd? (Age, Sex, Productivity etc)

____

____

____

(ii) If Yes in C.7 (i) above what camel classes are grouped together – (**use codes below**).

Group 1 – [______][_______][_______]

Group 2 – [______][_______][_______]

Group 3 – [______][_______][_______]

Group 4 – [______][_______][_______]

Group 5 – [______][_______][_______]

Codes

1 = Calves (less than one year)
2 = Young female (pre-breeding)
3 = Young male (pre-breeding)
4 = Adult female (breeding age)
5= Lactating animals
6 = Males (entire)
7 = Males (castrated)
8= Other

C.8. Where do you graze the following classes of camels?

**Class of camel**	**Most utilized grazing areas**		**Reasons**
	**In wet season**	**In dry season**	
1= Calves (less than one year)			
2= Young female (pre-breeding)			
3= Adult female (breeding age)			
4= Lactating animals			
5= Males (entire)			
6= Males (castrated)			

C.8.(i) Other than livestock keeping, do any of your household members have other sources of income?

1. Yes[________] 2. No[________]

(ii) If **YES** in (i),What are these other sources of income? **(Indicate all that apply) [____________]**

1=Farming	4=Business
2=Formal employment	5=Others (specify)___________________
3=Informal employment	

(iii) Please rank important economic activities that generate income for the household

Activity	Rank
Camel keeping	[______]
Other livestock species (specify)...............	[______]
Farming i.e. crop production	[______]
Formal Employment	[______]
Informal employment	[______]
Business	[______]
Others (specify)_____________________	[______]

D. Knowledge of camel trypanosomiasis and its management

D.1. What diseases affect camels in this area? (Respondents should be allowed to give local names)

____

____

____

____

____

D.2. (If **trypanosomiasis is listed**) how do you know that an animal is infected by trypanosomiasis? (Your observation to conclude that a camel is infected with trypanosomiasis*)*? *[Tick, √ if mentioned*]

1. Loses body condition[____________]
2. Develops a drooping hump [____________]
3. Unable to walk long distances [____________]
4. Sits down frequently
5. The camel develops swellings on the feet, underbelly and eyelids [____________]
6. The hair coat becomes rough [____________]
7. Produces a lot of saliva and tears
8. Shivers as if feeling cold [____________]

9. Does not feed well [___________]
10. Diarrhoeas [___________]
11. Produces less milk
12. Aborts if pregnant [___________]
13. Others, specify ____
14. ____
15. ____
16. ____
17. ____
18. ____

D.3. what causes trypanosomiasis in camels? [___________]

1= Tsetse fly   2= Biting Fly   3= Ticks   4= Mosquitoes 5= others (Specify)......................

D.4. (**If tsetse fly and biting flies are mentioned in D3 above**). When do you experience a heavy presence of the flies in your camel herd? [___________]

1= Rainy season 2 = Dry season 3= immediately after rainy season
4 = All seasons 5 = Other (Specify)...

D.5 How do you control the flies that cause camel trypanosomiasis? [___________]

1= Acaricide application   2= Avoid infested areas   3= Do nothing

4= Other (Specify)...

D.6.(i) When did you last experience a case of camel trypanosomiasis in your camel herd (**give month and year**)

[month____________ year__________________]

(ii) Which class (es) of animal in the herd was affected in this last case mentioned above (***use codes***)

[___________]

Codes

1 = Calves (less than one year)
2 = Young female (pre-breeding)
3 = Young male (pre-breeding)
4 = Adult female (breeding age)
5 = Males (entire)
6 = Males (castrated)

(iii). Did you treat the animal(s)?

1. Yes[_______] 2. No[_______]

(iv). IF YES, after how long did you take before you treated the camel after realising that it was sick? Give number of days - [___________________]

(v) What did you use to treat the animal?

1= Conventional drugs [_______]

2= herbal concoction [_______]

3= others [_______]

If conventional drugs used,

(vi) Which drug did you use? ***<u>give name or names if drugs were mixed</u>***

____

____

D.7 How did you prepare the drugs for administration and how did you administer it?

- ***Indicate Quantity of drug/s used***
- ***Indicate volume of water used***
- ***Indicate the mode of delivery- intravenous injection, muscle injection or oral***

____

____

____

D.8 What was the outcome after administering the treatment? [________________]

1= Camel recovered fully 2= Camel did not recover and died

3=Other (specify)...

D.9. More often than not, which classes of camels in your herd are most affected by trypanosomiasis. (**List in order of those mostly affected**) [______][______][______][______] (codes)

Codes for class

1 = Calves (less than one year)
2 = Young female (pre-breeding)
3 = Young male (pre-breeding)
4 = Adult female (breeding age)
5 = Males (entire)
6 = Males (castrated)

D.10 During treatment of trypanosomiasis in camels, do you normally mix different types of drugs together?

1. Yes[_______] 2. No[_______]

D.11. If YES which drugs do you mix and why?

____

____

____

D.12 How many times do you treat the sick camel for it to recover? [_________]

1= Once only 2.= twice only 3= Three times only 4= Others (specify)…………..

D.13 Do you think that available drugs are effective against trypanosomiasis in camels?

1. Yes[_______] 2. No[_______]

D.14 If **NOT**, what do you think is the reason(s)?

____

____

____

D.15 What do you **Mainly** do to prevent your animals from trypanosomiasis? [_______][_______]

1. = Spraying animals with suitable acaricide (***Name the acaricide***)……………………..)
2. = Avoiding vector infested areas
3. = Injecting them with preventive drugs
4. = Others, (Specify)...

D.16 Where do you **MAINLY** get your veterinary drugs from? (**List in order of importance**)

[_______][_______][_______][_______]

1. Agrovets (duka)
2. From open market, during market days
3. Hawkers on the streets
4. Drug companies
5. Government Veterinary Officer
6. Private vets.
7. Other (Specify)……………………………………………………………

D.17 (i) Is this where you obtained the drug you used to treat your last case of trypanosomiasis in your herd?[_____]

1. Yes[_______] 2. No[_______]

ii) Are drugs that you use to treat trypanosomiais always available in the outlet whenever you need them?

1. Yes[_______] 2. No[_______]

iii) What do you do when you fail to get the drugs you are looking for in the outlet?

____

____

D.18 More often than not, who treats your sick animals? [_____][_____]

1= Government Veterinary officers 2= Animal Health Workers
3= Self 4. Herders 5= Others (Specify)……………………………………………………….

D.19 ***(If farmer treats animals by himself ask)***, Do you normally experience any problems when treating your sick animals

1. Yes[_______] 2. No[_______]

D.20 If YES, What are the common problems encountered

____

____

D.21 (**If not mentioned above**) Is ethno-medicine used in treatment of camel trypanosomiasis in this community?

1. Yes[_______] 2. No[_______]

D.22. If yes please give the ethno medical materials/plants used in the treatment of *trypanosomiasis*

**Name of ethno medicinal drug/plant**	**Number of times drug used in last 12 months**	**Mains sources of the drug/substances**	**Do animals recover when drug is used**	**How do you prepare the drug before administering**

E. Details on camel Sales

E.1 Have you sold any camels during the last ONE year?

1. Yes[________] 2. No[________]

E.2 If yes above, how many camels did you sell ....

E.3 If camels were sold please give the following information

Name of market	Distance to the market from home	Class of animal (code)	Number of animals sold (last 12 months)
		[_______]	
		[_______]	
		[_______]	
		[_______]	
		[_______]	

Codes for class

1 = Calves (less than one year)
2 = Young female (pre-breeding)
3 = Young male (pre-breeding)
4 = Adult female (breeding age)
5 = Males (entire)
6 = Males (castrated)

E.4 What other management practices do you follow to improve the health and condition of camels before sale? (Indicate all that apply)

1= Deworming, 2= Breeding with improved breeds, 3= Feed supplementation,

4= Others (specify) ....

F. Camel inflows

F.1 Please tell us about the inflows of camels into your herd during the last 12 months:

Inflow type (code)	Month	Type of animal (code)	Number of animals	If purchased, where was it purchased (code)
[______]		[______]	[_______]	[_______]
[______]		[______]	[_______]	[_______]
[______]		[______]	[_______]	[_______]
[______]		[______]	[_______]	[_______]
[______]		[______]	[_______]	[_______]
[______]		[______]	[_______]	[_______]
[______]		[______]	[_______]	[_______]

Codes

**Type of inflow**	**Type of animal**
1= Birth<br>2=Purchase<br>3=Gift in<br>4= Exchange in<br>5=Others (specify)______________________________	1= Kid/lamb (<5 months)<br>2= Young female (pre-breeding)<br>3= Adult female (breeding age)<br>4= Males (entire)<br>5= Males (castrated)<br>6= Old female animals
**Where Purchased**<br>1=Farm gate<br>2=Village market<br>3=Distant market<br>4=Local business centre<br>5=Local dip<br>7=Auction<br>8=Livestock trader<br>9=Other (specify)__________________________	

G. Other assets owned by the household and household composition

G.1 Do you own the following assets?

Type of asset		Does the farmer own the asset? 1= Yes 2=No	Number owned
Rental buildings			
Vehicles	Lorries		
	Pick-ups		
	Tractors		
	Cars		
	Motor cycles		
Bicycles			
Animal pulled carts			
Radios			
TV			
Cell phone			
Other assets (specify)			

G.2. Please provide the following information about the members of your household

i. Number of spouses____
ii. Number of children____
iii. Number of siblings____
iv. Others ____

Thank you
