# Supplementary material for: Variation of sensitivity of Trypanosoma evansi isolates from Isiolo and Marsabit counties of Kenya to locally available trypanocidal drugs
Source: PLoS One. 2023 Feb 2;18(2):e0281180. doi: 10.1371/journal.pone.0281180 (PMC9894490; doi:10.1371/journal.pone.0281180)
Supplement: S2 Appendix — (PDF) [file pone.0281180.s002.pdf]

S2 Appendix. Clinical signs the camel herders associated with camel surra

Item			Community orientation			
Item	Somali (N=111)	Rendille (N=69)	Gabbra (N=84)	Borana (N=35)	Turkana (N=41)	Total (N=340)
Clinical sign mentioned:						
i. Loss of body condition	94 (84.7)	65 (94.2)	79 (94.0)	30 (85.7)	40 (97.6)	308 (90.6)
ii. Drooping hump	31 (27.9)	38 (55.1)	46 (54.8)	3 (8.6)	21 (51.2)	139 (40.9)
iii. Inability to walk long distances	78 (70.2)	49 (70.0)	50 (59.2)	20 (57.1)	25 (61.0)	222 (65.3)
iv. Swelling of feet, under belly and eye lids	59 (53.1)	25 (36.2)	20 (23.8)	9 (25.7)	20 (48.9)	133 (39.1)
v. Sitting down frequently	78 (70.2)	34 (49.3)	33 (39.3)	20 (57.1)	28 (68.3)	193 (56.8)
vi. Rough hair coat	86 (77.5)	56 (81.2)	70 (83.3)	18 (51.4)	30 (71.2)	260 (76.5)
vii. Lacrimation	72 (64.9)	33 (47.8)	29 (35.7)	24 (68.6)	31 (75.6)	189 (55.6)
viii. Shivering	80 (72.1)	30 (43.5)	57 (67.9)	19 (54.3)	9 (22.0)	195 (57.4)
ix. Inability to feed well	86 (77.5)	43 (62.3)	65 (77.4)	27 (77.1)	26 (63.4)	247 (72.6)
x. Diarrhoea	48 (43.2)	34 (49.3)	18 (21.4)	7 (20.0)	14 (34.1)	121 (35.6)
xi. Reduced milk production in lactating females	78 (70.3)	37 (53.6)	55 (65.5)	28 (80.0)	27 (65.9)	225 (66.2)
xii. Abortion in pregnant females	75 (67.6)	46 (66.7)	55 (65.5)	18 (51.4)	18 (43.9)	212 (65.3)
